# Analytical methods for quantitating sulfate in plasma and serum

**DOI:** 10.1042/EBC20230092

**Published:** 2024-12-04

**Authors:** Prasidhee Vijayakumar, Paul A. Dawson

**Affiliations:** Mater Research Institute, The University of Queensland, Woolloongabba QLD, Australia

**Keywords:** Assay, Biochemical pathology, Method, Sulfataemia, Sulfate

## Abstract

Circulating sulfate needs to be maintained at sufficiently high levels for healthy growth and development. Animal studies have shown the adverse physiological consequences of low circulating sulfate level on the skeletal, neurological and reproductive systems. However, sulfate is not routinely measured in clinical investigations, despite the importance of sulfate being documented over the past several decades. Several methods have been developed for measuring serum and plasma sulfate level in animals and humans, including a range of barium sulfate precipitation techniques that have been a major focus of sulfate analytics since the 1960s. Evaluation of an ion chromatography method demonstrated its utility for investigation of sulfate levels in human health. More recently, liquid chromatography-tandem mass spectrometry has been used to show hyposulfatemia in a human case of mild skeletal dysplasia. This article provides an overview of analytical methods for measuring sulfate in serum and plasma, highlighting the strengths and limitations of each method.

## Introduction

**Figure 1:**
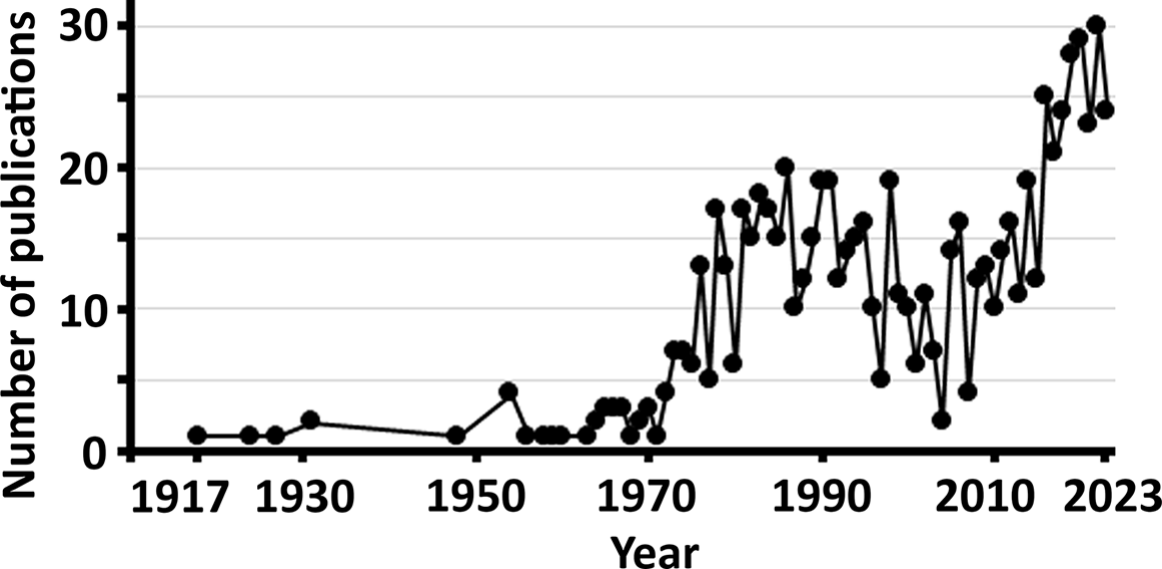
The number of articles published in the field of circulating inorganic sulfate level Articles were identified in PubMed 7 January 2024 using the search term (inorganic) AND ((sulfate) OR (sulphate)) AND ((blood) OR (plasma) OR (serum)). The increasing number of articles in recent years reflects the current interest in the physiological importance of circulating sulfate level in mammalian physiology.

**Figure 2:**
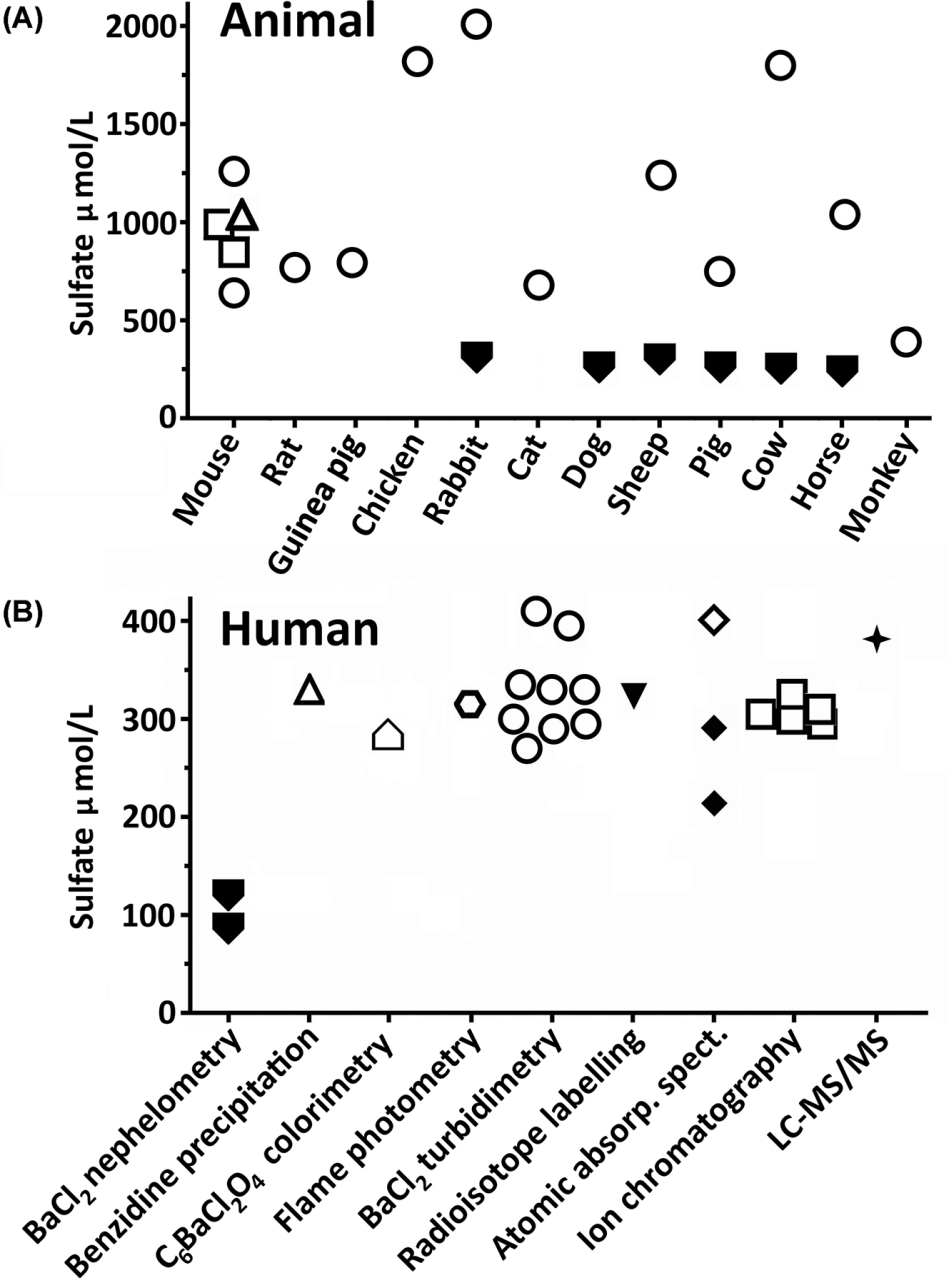
Inorganic sulfate levels in plasma and serum (**A**) Animal [2,3,15,19,20] and (**B**) human [2,13,15–19,21–32] plasma (*filled symbols*) and serum (*unfilled symbols*) levels (average of each study) determined using the methods shown at the bottom (*matching symbols for panels A and B*).

More than 100 years ago, biochemists started to measure inorganic sulfate in human and animal blood samples using a method to precipitate sulfate with barium chloride (BaCl_2_) [1,2]. Those early studies identified sulfate to be the fourth most abundant anion in circulation. Since then, several methods for measuring serum and plasma sulfate level in research settings have been reported. This field of inorganic sulfate research has been sporadic up until the turn of the century (Figure 1) when methodologies were being developed and primarily used to measure sulfate levels in a range of animal and human serum and plasma samples (Figure 2A,B). More recently, animal studies have shown the adverse physiological consequences of low circulating sulfate levels [3–7]. Those findings have led to an increased interest in sulfate analytics for the investigation of atypical sulfate levels in human health and disease [8–13]. To pave the way for routine sulfate testing, certain methods have been validated for their analytical utility in clinical settings [14]. To acknowledge the advantages and limitations of each methodology, this article provides an overview of methods that have been used to quantitate sulfate in plasma and serum.

## Sulfate precipitation procedures

BaCl_2_ added to solutions containing inorganic sulfate, leads to the precipitation of barium sulfate (BaSO_4_) which can then be analyzed by measuring the intensity of light that is scattered (nephelometric) or transmitted (turbidimetric) through the samples. Early studies used BaCl_2_ nephelometry to measure plasma sulfate levels in animals (Figure 2A) and humans (Figure 2B) [15]. However, that methodology was abandoned as it underestimated sulfate levels when compared with BaCl_2_ turbidimetry (Figure 2A,B). More recently, indirect methods that measured excess barium after barium sulfate precipitation of human plasma or serum samples using flame photometry [16], atomic absorption spectrophotometry [17] or radioisotope labelling [18] were also abandoned as they were more time-consuming than the turbidimetric method which produced similar findings (Figure 2B). Benzidine precipitation of sulfate has also been used to measure mouse (Figure 2A) and human (Figure 2B) serum sulfate levels [19]. This method gave similar results to BaCl_2_ turbidimetry but has been discontinued due to its carcinogenic potential.

BaCl_2_ turbidimetry has been the most extensively used method for the quantitation of sulfate in serum samples from animals [2,3,15,19,20] and humans [2,13,15–19,21–32] (Figure 2A,B). The development of commercially available kits (Sigma-Aldrich, BioAssay Systems, Abcam) which are based on this methodology, highlights a growing interest in sulfate analytics. Human serum sulfate levels measured via BaCl_2_ turbidimetry are approximately 300 μmol/L (Figure 2B), whereas levels in animals range from 390 μmol/L in monkeys to 2010 μmol/L in rabbits (Figure 2A), which is an approximate 7-fold difference in sulfate levels among species. This broad range in serum sulfate levels also demonstrates the utility of the BaCl_2_ turbidimetric method for quantitating high serum sulfate levels that can occur in certain human physiological conditions, including pregnancy [10], hyperthyroidism [33], pregnancy-induced hypertension [34] renal glomerular dysfunction [33] and renal failure [35]. However, the assay becomes prone to variable error at the lower limit of normal levels [33].

The BaCl_2_ turbidimetric assay has been modified several times to improve both precision and reproducibility, with key criteria being: (i) acidic conditions are needed to avoid interference from phosphate [36]; (ii) serum and plasma needs to be deproteinized, usually via vortex-mixing with trichloroacetic acid, to prevent interference of proteins in the transmitted light [37]; (iii) wavelength of 600 nm minimizes interference from colored compounds in the sample [38,39]; and (iv) suspension of the BaSO_4_ precipitate needs to be enhanced by premixing BaCl_2_ with stabilizing reagents, such as gelatin [21], glycerol [40], dextran [41], polyethylene glycol [38] or agarose [37], with the latter two compounds providing the most linear standard curves. Early studies required milliliter volumes of whole blood to quantitate serum and plasma sulfate levels using the BaCl_2_ turbidimetric assay [37]. When blood volume is limited (i.e. small animal research), a scaled-down version of the assay can be performed using flat-bottom well microtiter plates and a microplate spectrophotometer [42].

Barium chloranilate (C_6_BaCl_2_O_4_) precipitates sulfate as BaSO_4_ and releases chloranilate which can be measured via colorimetric changes to the solution. In one study, human serum sulfate level determined by this method [32] was similar to those measured using the BaCl_2_ turbidimetric assay (Figure 2B). A benefit of using C_6_BaCl_2_O_4_ is that this method can measure sulfate level in the presence of sulfated glycosaminoglycans (i.e. heparin anticoagulant) which can interfere with BaCl_2_ precipitation [36]. However, subsequent investigations showed that slight changes in ionic concentrations of serum samples led to interference with the barium chloranilate/sulfate reaction, making the results unreliable [43]. Accordingly, C_6_BaCl_2_O_4_ is rarely used in the quantification of sulfate in serum and plasma samples.

## Ion chromatography

The use of ion chromatography to measure sulfate in clinical pathology settings was first reported in 1976 [44]. In comparison with barium precipitations methods, ion chromatography provided increased specificity, sensitivity and precision for the quantitation of serum sulfate [22]. The method also enabled the simultaneous determination of other anions in serum and a range of biological fluids, including cerebrospinal fluid and sweat [45]. The suppression ion chromatography and conductimetry method was quickly recognized as the reference method for the reliable determination of serum sulfate level (Figure 2B) [29,46,47]. Variations of the ion chromatography method have also measured human serum sulfate levels by incorporating UV detection (307 ± 92, *n*=20) [26] and conductimetry (290 ± 60, *n*=22) [48], which are comparable to data from the suppression ion chromatography and conductimetry method shown in Figure 2B.

More recently, the analytical utility of the suppression ion chromatography and conductimetry method for quantitating serum and plasma sulfate in clinical settings was reported [14]. That study showed: linearity of detection (0–1041 μmol/L), limit of detection 9 μmol/L, limit of quantification 27 μmol/L, as well as similar results for plasma and serum sulfate that were stable at ambient temperature for 1 day and at 4°C or −20°C for up to 30 days. The validated ion chromatography method showed robust lack of interference of other anions, including chloride which accounts for more than 90% of the free anionic charge in serum. This method has now been used to determine reference intervals for plasma sulfate in pregnant women at 10–20 (305–710 μmol/L) and 30–37 (335–701 μmol/L) weeks gestation, as well as in term venous cord plasma (175–603 μmol/L) [10]. These findings highlight the increased (up to 2-fold) circulating sulfate level during pregnancy, which is proposed to provide a reservoir of sulfate for the developing fetus [49]. The generation of sulfate reference ranges provides an important step towards understanding the pathophysiology of hyposulfatemia which is an emerging area of clinical interest. In particular, hyposulfatemia has been linked to the human and mouse *SLC13A1* sulfate transporter gene which is primarily expressed in the renal proximal tubule where it mediates sulfate reabsorption [3,50].

## Liquid chromatography-tandem mass spectrometry (LC-MS/MS)

LC-MS/MS has been used extensively for the quantitation of sulfated endogenous and exogenous compounds in serum [51], and is now emerging as an alternative tool for the determination of inorganic sulfate levels in healthy and diseased states. Recently, LC-MS/MS was used to confirm hyposulfatemia (65 μmol/L) in a patient with a homozygous loss-of-function *SLC13A1* variant and unexplained skeletal dysplasia [13]. In that study, the LC-MS/MS quantitation of control group serum sulfate level (381 μmol/L, mean) was higher than sulfate levels measured by ion chromatography for other control groups (Figure 2B). This may be due to age differences between the control groups, with higher serum sulfate levels reported in infancy and old age but lower for children [52]. Further evaluation of LC-MS/MS for the quantification of serum sulfate is needed to inform comparative analyses with other sulfate analytics, particularly ion chromatography.

## Strengths and limitations

As outlined above, numerous methods have been developed for the determination of sulfate level in plasma and serum samples. Many of these approaches have been discontinued due to toxicity concerns, the time-consuming nature of the procedure or unreliability of the test results, whereas other methods, including the BaCl_2_ turbidimetric assay, ion chromatography and LC-MS/MS are used in current research settings, albeit each with strengths and limitations (Table 1). Alternative technologies, that make use of molecular receptors [53] or fluorescent proteins fused to sulfate binding protein [54] offer the potential for new approaches towards sulfate determination in biological samples, including serum and plasma.

**Table 1:** Strengths and limitations of sulfate analytics

Method	Strengths	Limitations
BaCl_2_ nephelometry	Rapid, cost-effective	underestimates level
Benzidine precipitation	Specificity, sensitivity, precision	Carcinogenic
Barium chloranilate colorimetry	Rapid, cost-effective	Unreliable varying ion concentration
Flame photometry	Specificity, sensitivity, precision	Time-consuming
BaCl_2_ turbidimetry	Rapid, cost-effective	Sensitivity at low concentration
	Precision at high concentration	Specificity
Radioisotope labelling	Specificity, sensitivity, precision	Time-consuming
AAS	Specificity, sensitivity, precision	Time-consuming
Ion chromatography	Specificity, sensitivity, precision	Specialized equipment
	Determination of other anions	Cost
	Range of biological fluids	
LC-MS/MS	Specificity, sensitivity, precision	Specialized equipment
	Determination of other anions	Limited sulfate data
	Range of biological fluids	Cost

Abbreviations: AAS, atomic absorption spectrophotometry; LC-MS/MS, liquid chromatography-tandem mass spectrometry.

## Conclusion

The importance of sulfate in human physiology cannot be overestimated. Animal studies have shown the adverse consequences of hyposulfatemia on skeletal, neurological and reproductive biology. Given that sulfate biology is highly conserved across mammalian species, it is not surprising that hyposulfatemia is now being linked to adverse human health conditions. To fully appreciate the extent of sulfate deficiency in human health and disease, sulfate analytics need to be incorporated into routine clinical investigations.

## Summary

- Serum and plasma sulfate levels are not routinely measured in clinical investigations.
- Abnormally low serum and plasma sulfate levels are linked to adverse phenotypes in animals, affecting skeletal, neurological and reproductive systems.
- Emerging human research suggests that low circulating sulfate levels also lead to adverse physiological conditions.
- This article outlines methods used to measure sulfate, highlighting the strengths and limitations of each technique.

## Open Access

Open access for this article was enabled by the participation of University of Queensland in an all-inclusive *Read & Publish* agreement with Portland Press and the Biochemical Society under a transformative agreement with CAUL.

## Abbreviations

AAS: atomic absorption spectrophotometry
LC-MS/MS: liquid chromatography-tandem mass spectrometry
